# Ketone Body Metabolism in the Ischemic Heart

**DOI:** 10.3389/fcvm.2021.789458

**Published:** 2021-12-07

**Authors:** Stephen C. Kolwicz

**Affiliations:** Heart and Muscle Metabolism Laboratory, Department of Health and Exercise Physiology, Ursinus College, Collegeville, PA, United States

**Keywords:** ischemia, reperfusion, hypoxia, beta-hydroxybutyrate, ketosis

## Abstract

Ketone bodies have been identified as an important, alternative fuel source in heart failure. In addition, the use of ketone bodies as a fuel source has been suggested to be a potential ergogenic aid for endurance exercise performance. These findings have certainly renewed interest in the use of ketogenic diets and exogenous supplementation in an effort to improve overall health and disease. However, given the prevalence of ischemic heart disease and myocardial infarctions, these strategies may not be ideal for individuals with coronary artery disease. Although research studies have clearly defined changes in fatty acid and glucose metabolism during ischemia and reperfusion, the role of ketone body metabolism in the ischemic and reperfused myocardium is less clear. This review will provide an overview of ketone body metabolism, including the induction of ketosis via physiological or nutritional strategies. In addition, the contribution of ketone body metabolism in healthy and diseased states, with a particular emphasis on ischemia-reperfusion (I-R) injury will be discussed.

## Introduction

Ketone body metabolism has become an important topic in the scientific and medical communities over the last several years. The identification of elevated ketone body oxidation in hypertrophied and failing hearts (1, 2) and the potential of exogenous ketone body supplements to promote exercise performance (3) have been a critical driver in research. Since ketone bodies are suggested to be more energy efficient than glucose or fatty acids (4, 5), the utilization of ketone bodies as an energy source may be advantageous for failing myocardium or exercising skeletal muscle. Certainly, the therapeutic and ergogenic potential of nutritional or pharmacological strategies that promote cardiac and muscular ketone body metabolism are of increased interest.

Ketogenic diets (KD) are high fat, low carbohydrates that were originally developed for the treatment of epilepsy (6). KDs can also be an effective treatment for inherited metabolic disorders of glucose metabolism (7). Although KDs appear to be effective strategies for weight loss, at least in the short-term, their ability to promote exercise performance appears to be limited (8). The use of exogenous ketone body supplementation has been of increased interest in exercise and sport performance, but the effectiveness of supplements remains controversial (9, 10). Although the KD or supplementation strategies appear to be recognized as plausible treatments in models of cardiac dysfunction and failure (11), there is less discussion regarding the role of ketone body metabolism in ischemic heart disease.

Research studies have uncovered critical changes in cardiac substrate metabolism under conditions of stress, especially in pathological hypertrophy (12, 13), heart failure (14, 15), ischemia-reperfusion (16–18), and obesity/diabetes (19, 20). Although the importance of ketone body metabolism to the hypertrophied and failing heart has been well-discussed, the contribution of ketone body oxidation to the myocardium following ischemia has received less attention. In this review, ketone body metabolism in the healthy and diseased myocardium will be discussed, with a particular focus on the impact of ketone body metabolism in the functional recovery from ischemic injury.

## Metabolic Pathways of Ketone Bodies

**Figure 1:**
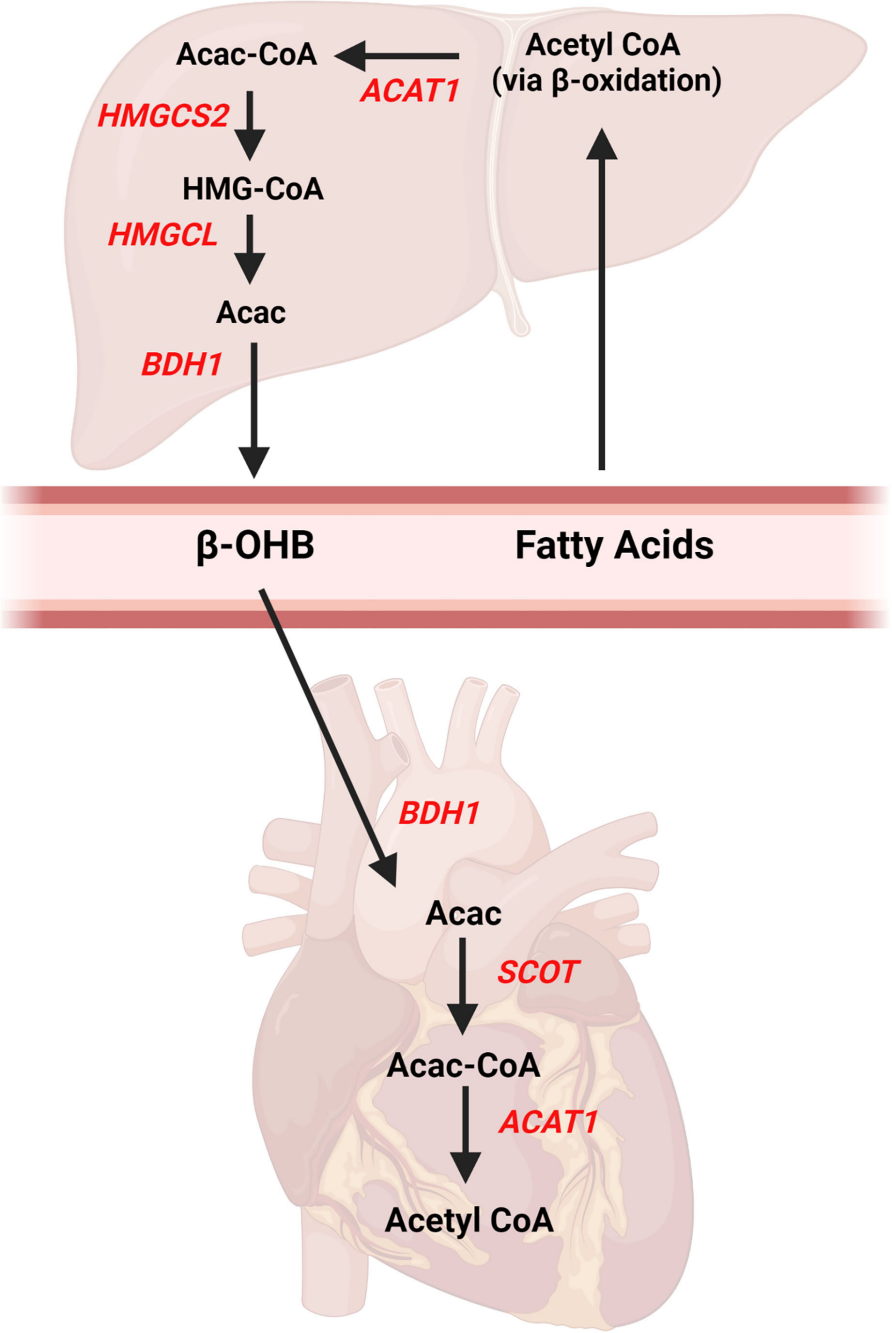
Ketone body pathways in the liver and heart. Ketogenesis occurs primarily in the liver from fatty acids obtained from the blood. Acetyl CoA is formed from hepatic beta-oxidation and ultimately converted to beta-hydroxybutyrate (β-OHB) via multiple enzymatic reactions. Once β-OHB enters the heart, a series of reactions forms Acetyl CoA for entry into Kreb's Cycle. AcAc, acetoacetate; Acac CoA, acetoacetyl CoA; ACAT1, acetyl-coenzyme A acetyltransferase 1, βOHB, beta-hydroxybutyrate; BDH1, mitochondrial beta-hydroxybutyrate dehydrogenase; HMGCS2, 3-hydroxy-3-methylglutaryl-CoA synthase 2; HMGCL, HMGC-CoA lyase; SCOT, succinyl-CoA:3-oxoacid-CoA transferase. Created with BioRender.com.

Ketone bodies are 4-carbon molecules that are synthesized through the process known as ketogenesis and broken down and utilized within tissues via the ketolysis pathway (also referred to as ketone body oxidation). Three ketone bodies exist: acetone, acetoacetate, and beta-hydroxybutyrate (β-OHB). β-OHB is the ketone body which is in highest concentration in the blood and is typically measured in many commercially available assay kits or handheld meters used in the research setting. The liver is the primary site of ketogenesis (Figure 1), which generates acetyl-CoA via beta-oxidation of fatty acids. The acetyl-CoA is ultimately converted into acetoacetone or β-OHB via reactions that require several enzymes, namely mitochondrial acetyl-CoA acetyltransferase 1 (Acat1, commonly referred to as thiolase), 3-hydroxy-3-methylglutaryl-CoA synthase (Hmgcs2), HMGC-CoA lyase (Hmgcl), and mitochondrial beta-hydroxybutyrate dehydrogenase (Bdh1). Of note, acetoacetone is generated as a result of the Hmgcl reaction and can be converted to β-OHB via Bdh1. Once β-OHB enters peripheral tissues via monocarboxylate transporters, MCT1 (encoded by the Slc16a1 gene) or MCT2 (encoded by the Slc16a7 gene) (21), the ketolytic process commences (Figure 1). Acetyl CoA is produced through rapid oxidation of β-OHB by Bdh1, succinyl-CoA:3-oxoacid-CoA transferase (Scot, encoded by the Oxct1 gene), and Acat1, which then is available for entry into the tricarboxylic acid (TCA) cycle. These ketolytic enzymes are present in the heart and the reactions are primarily based on the substrate availability. However, the Scot reaction is also dependent upon the availability of succinyl CoA.

Older texts often discussed the presence of ketone bodies as negative consequences of abnormal metabolic processes (22, 23). However, since serum ketone bodies are measurable in the fed and fasted condition of healthy humans and animals, there must be some metabolic value. In fact, the use of ketone bodies as a metabolic fuel is suggested to be more energy efficient (4, 5). However, the total ATP yield per carbon atom for ketone bodies is slightly above glucose (~5.4 vs. ~5.2), while both substrates lag behind fatty acids (~6.7) (24, 25). In terms of ATP per oxygen (i.e., P/O ratio), ketone bodies are slightly lower than glucose (~2.50 vs. ~2.58), with both yielding higher ratios than fatty acids (~2.33) (24, 25). Based on these values, ketone bodies would rank second on both scales in relation to glucose and fatty acids. However, in isolated perfused heart experiments in rodents, the presence of ketone bodies in the perfusate increased cardiac efficiency (26, 27) and ATP production (28). Therefore, the relationship of ketone bodies to overall cardiac metabolism may require more intricate examination.

## Physiological and Nutritional Ketosis

Ketosis is defined as a physiological condition where serum ketone body concentrations are elevated acutely. Studies in humans and rodents commonly report serum ketone body concentrations in the range of ~0.1 to 0.5 mM, so ketosis is generally identified as serum concentrations above 0.5 mM. Conditions that result in nutrient deprivation or low glucose availability, such as fasting/starvation and exercise (29, 30), are commonly associated with elevated serum ketone body concentrations, which is termed “physiological ketosis.” Diabetics, particularly Type I diabetics, may also exhibit elevated serum ketone body levels (31, 32). However, when in the 3.8–25.0 mM range and accompanied by low arterial pH values, the term “ketoacidosis” is used to reflect a potentially dangerous pathological state (33–35). More recently, the term “nutritional ketosis” has been used to better identify a state where ketosis was induced by nutritional or supplementation strategies (36–38). The use of these specific terms could help differentiate between the physiological/pathological and/or intentional/unintentional causes of ketosis.

In our recent study, we explored physiological ketosis in response to both fasting and endurance exercise (29). Female mice fasted up to 8 h, demonstrated gradual increases in serum ketone body concentrations, which peaked at ~ 0.8 mM (29). Our unpublished data showed that male mice respond similarly, with peak serum ketone body concentrations of ~0.7 mM. In response to endurance exercise of up to 2.5 h, serum ketone body levels rose to ~1.2 mM in female mice (29). Interestingly, this “exercise-induced ketosis” was blunted in fasted female mice (29). These values of physiological ketosis related to fasting or exercise are fairly consistent across the literature in both humans (39–41) and rodents (30, 42, 43). However, whether physiological ketosis is consistent between the sexes is relatively unexplored.

To induce nutritional ketosis, one strategy involves the consumption of the ketogenic diet (KD), a diet that is typically low in carbohydrates with high fat and adequate protein. The KD was originally used as a treatment for individuals with diabetes, epilepsy, and other neurological conditions approximately 100 years ago (6, 44, 45). This classical KD called for a fat to protein plus carbohydrate ratio of 4:1 (46), which typically results in a dietary intake of 80-90% of calories from fat, 10-15% calories from protein, and <5% of calories from carbohydrate sources (5). Although the use of the KD in research has increased significantly over the last several years, the dietary composition can deviate quite substantially from the classical KD. In some human studies, dietary regimens referred to as low-carbohydrate KD (LCKD) or very low-carbohydrate KDs (VLCKD) are used with carbohydrates ranges from ~10% to 45% of total calories (47–50). Despite the varied ranges of carbohydrate intake, all of these diets tend to be included in the KD category. Therefore, careful attention to the details of the dietary composition is needed to adequately interpret experimental findings.

Although the KD has been successful for weight reduction in mice (30, 51) and humans (52, 53), the high fat and low carbohydrate intake may be problematic for long-term adherence. Moreover, KDs are associated with negative consequences such as abnormal glucose homeostasis (54–56), hyperlipidemia (29, 54, 57, 58), and liver steatosis (29, 55). Therefore, another strategy to induce nutritional ketosis has been developed, which involves exogenous ingestion of ketone bodies as a nutritional supplement. These ketone body supplements are now commercially available and have been tested in several studies evaluating the potential ergogenic aid on exercise performance (3, 59–63). However, special attention must be paid to the exact formulation of these supplements as they are available in ketone salts (KS) or ketone esters (KE). Although more readily available and inexpensive, the use of KS supplements can be problematic, depending on the exact formulation, which may include β-OHB or 1,3-butandeniol (BD). In the salt form, β-OHB is present in both the active (D) and non-metabolizable (L) form, which may result in a less effective increase in serum ketone bodies (64, 65). KS supplements that contain BD may also be a less desirable method to induce ketosis since dehydrogenase enzymes in the liver are required to create β-OHB (64, 66). Although several formulations of KE supplements are available, the (R)-3-hydroxybutyl (R)-3-hydroxybutyrate ketone monoester, which converts to D-β-OHB and BD (67) has become the most commonly used. This KE, when combined with carbohydrates, elicited an increase in exercise performance in trained cyclists (3). Of note, other studies using KE supplementation have not consistently shown an increase in exercise performance (61–63).

## Ketone Body Metabolism in the Healthy Heart

The term, metabolic flexibility, has become synonymous with the cardiometabolic profile of the healthy heart. Due to the enormous requirement for continual replenishment of energy stores, the heart must possess an ability to utilize any carbon-based substrate available. To this end, the heart can metabolize the exogenous substrates fatty acids, glucose, lactate, amino acids, and ketone bodies to produce energy. Research suggests that the normal healthy heart generates approximately 60-80% of its energy requirements from fatty acids, with approximately 10-20% from glucose (68–70). Lactate can also be an important fuel, particularly during instances of increased workload, such as exercise (71, 72). The contribution of amino acids to cardiac energy metabolism is reported to be low, ~1-3% (73, 74). However, this low contribution to oxidative metabolism should not be interpreted as insignificant as disruption of amino acid catabolism in genetic models can be detrimental, particularly in stressed conditions (75).

Although previously considered to be a relatively minor substrate, ketone body oxidation is reported to contribute ~10-20% to cardiac energy metabolism (28, 43). Interestingly, ketone body oxidation may not be essential to the healthy myocardium as cardiac-specific deletion of Bdh1 (76) or Scot (Oxct1) (77) virtually eliminates ketone body oxidation with no myocardial phenotype in unstressed conditions. In contrast, cardiomyocyte overexpression of Bdh1 increases ketone body oxidation by approximately 70% without negatively affecting cardiac function (78). These studies in transgenic mice would suggest that low or high ketone body oxidation is relatively inconsequential to the heart in unchallenged situations. However, a potential concern is the effect of changes in ketone body availability and delivery on the usage of other fuels, particularly glucose and fatty acids, on cardiac metabolism. Indeed, increased delivery of β-OHB suppresses fatty acid oxidation but does not significantly affect glucose oxidation in animal models (30, 76, 79). Increased ketone bodies also decreased myocardial glucose uptake in humans (80). However, increasing β-OHB concentrations in working heart preparation in mice did not affect fatty acid oxidation and tended to enhance glucose oxidation (28). In relation to this, increasing glucose uptake via overexpression of glucose transporter 4 (Glut4) suppressed ketone body oxidation in isolated perfused mouse hearts (81). These seemingly discrepant findings are likely due to differences in the experimental logistics, particularly in the concentrations of ketone bodies employed. However, the influence of changes in ketone body oxidation on oxidation of other substrates appears to be essentially meaningless, as cardiac function remains relatively normal in these situations, thus highlighting the metabolic flexibility of the heart under normal, baseline conditions.

## Ketone Body Metabolism in the Obese and Diabetic Heart

The discussion of ketone bodies in settings of obesity and diabetes are traditionally thought to represent a pathophysiological state due to the condition of diabetic ketoacidosis (DKA) that can result in serious clinical complications (33–35). Indeed, Type 1 diabetic (T1D) patients without insulin therapy quickly develop ketonemia within 5 h (32). Therefore, the American Diabetes Association recommends regular monitoring of both glucose and ketone bodies for T1D (82). However, blood levels of ketone bodies are also increased in Type 2 diabetics (T2D) patients (31), indicating that systemic disruptions in glucose homeostasis, either via insulin deficiency or insulin resistance, result in changes in systemic ketone body concentrations. Whether the elevation in ketone body availability contributes directly to the cardiac dysfunction often associated with the obese and diabetic heart is not known.

The cardiometabolic profile of the diabetic and obese heart has been generally described as increased reliance on fatty acid oxidation with a concomitant reduction in glucose oxidation (83), which in is contrast to the hypertrophied and failing heart discussed in the next section. However, studies conducted over the last several years also demonstrate alterations in cardiac ketone body metabolism in diabetes. In T2D patients, myocardial uptake of the ketone bodies, β-OHB and acetoacetate were higher than non-diabetic controls, inferring a possible increase of ketone body oxidation in human hearts (84). However, ketone body oxidation was shown to be reduced in isolated hearts from diabetic (*db/db*) mice (20). Consistent with this, T1D, induced by streptozotocin (STZ), or T2D, induced by 12-weeks of high fat diet, was associated with reduced mRNA expression and protein level of myocardial Bdh1 (81). Moreover, STZ hearts demonstrated accumulation of cardiac β-OHB with a tendency for reduced myocardial β-OHB oxidation (81). One potential interpretation of these animal studies is that the reduction in ketone body oxidation is a hallmark of the diabetic heart, which might serve as a therapeutic target. In support of this, a recent study demonstrated improved systolic and diastolic function as well as increased expression of ketone body oxidation enzymes in *db/db* mice fed a diet supplemented with KE (85). Future research can help to clarify the impact of enhanced ketone body metabolism in the obese and diabetic myocardium.

## Ketone Body Metabolism in Hypertrophy and FailurE

Over the last 5 years, the importance of cardiac ketone body metabolism in the pathological setting has gained great attention (1). Although the metabolic derangements that occur in the hypertrophied and failing heart, including significant decreases in fatty acid oxidation concomitant with increased glycolysis, are well established (14, 69, 86), the role of ketone body oxidation previously received little focus. However, seminal studies in humans (87) and mice (88) have underscored the contribution of ketone body metabolism to cardiac pathologies, particularly in heart failure. These publications have led to a host of recent studies that have examined the role of ketone body metabolism in cardiac disease (28, 76, 78, 85, 89, 90).

Findings from recent studies have indicated that the hypertrophied and failing heart demonstrates elevated ketone body oxidation (76, 87, 88), which provides an alternative fuel source to maintain cardiac function. These studies have provided the framework for targeting ketone body metabolism as a potential therapeutic treatment for cardiac dysfunction. Indeed, cardiac-specific deletion of Bdh1 in mice exacerbates cardiac dysfunction and remodeling in a heart failure model (76), while overexpression of Bdh1 protects against cardiac dysfunction and adverse myocardial remodeling (78). These provocative findings could support the use of the KD as a nutritional strategy to treat heart failure. In support of this idea, mice fed a KD of ~80% fat, ~20% protein, ~0% carbohydrates for 4-weeks had reduced pathological remodeling in a combined pressure-overload/myocardial infarction heart failure model (76). However, long term KD treatment (i.e., 62 weeks) may worsen diabetic cardiomyopathy in rats (89). Considering the high fat content of KDs, this may not be a suitable long-term strategy so ketone body supplementation might offer a better alternative. In agreement with this notion, a diet supplemented with KEs improved cardiac function, in part by increased mitochondrial oxygen consumption, in type 2 diabetic mice (85). However, although β-OHB infusion has been reported to increase cardiac function in failing canine hearts (76), provision of β-OHB in the perfusate to hypertrophied and/or failing mouse hearts in isolated heart experiments may not improve cardiac efficiency (28) or myocardial energetics (90). Certainly, experimental conditions, including *in-vivo* vs. *ex-vivo* situation and the concentration of ketone bodies used, are a concern.

## Ketone Body Metabolism During Ischemia and Reperfusion

Consistent with the cardiac metabolic profile in hypertrophied and failing hearts, the research literature has clearly defined changes that occur during ischemia-reperfusion (I-R). During reperfusion following ischemia, excessive rates of fatty acid oxidation (16, 18) and uncoupling of glycolysis from glucose oxidation (17, 91) have been reported, which may contribute to the reduced functional recovery from ischemic injury. In support, stimulation of glucose oxidation (18, 92, 93) or inhibition of fatty acid oxidation (94–96) improves functional recovery after cardiac ischemia. Oxidation of ketone bodies is suggested to supplant fatty acid oxidation (30, 76, 79), and as a result, upregulation of ketone body metabolism may decrease reliance on fatty acid oxidation and/or improve the coupling of glycolysis to glucose oxidation. Therefore, investigations examining the relationship of the ketogenic diet, ketone body metabolism, and functional recovery from ischemia are needed.

Although the role of ketone body metabolism in heart failure has gained significant attention in the research and medical communities as a therapeutic intervention (2, 97), the role of ketone body metabolism in the ischemia and post-ischemic heart has been less discussed. However, a recent publication identified that circulating ketone bodies were significantly elevated in patients presenting with ST-Segment Elevation Myocardial Infarction (STEMI) (98). Interestingly, elevations in ketone body concentrations 24 h after percutaneous coronary intervention (PCI) were independently associated with greater infarct size and lower LV ejection fraction after 4-months (98). β-OHB concentrations were higher in blood from acute MI patients and mice following left anterior descending (LAD) ligation surgery, which was negatively associated with left ventricular ejection fraction in both models (99). Ketone bodies, particularly β-OHB, was associated with higher odds ratios for myocardial infarction and ischemic stroke (100). Increased ketone body concentrations have also been reported in heart failure patients (87, 101, 102), after fasting and exercise (29, 30), and diabetes (31, 32). Therefore, speculation could arise as to whether elevations in serum ketone body concentrations are a hallmark of metabolic stress.

Since ischemia represents a condition with low oxygen and nutrient availability, the likelihood that ketone bodies contribute to metabolic processes would be quite low. Consistent with this, a recent report demonstrated that ketone body utilization was suppressed by myocardial ischemia in patients presenting with chest pain (103). However, β-OHB has been shown to accumulate in rat hearts exposed to low-flow ischemia (104), and since the perfusate was devoid of ketone bodies, this suggests that ketogenesis was active in the ischemic heart. Supporting this, inhibition of Hmgcs2 decreased β-OHB accumulation and improved functional recovery during reperfusion (104). This reported finding is somewhat curious, as extrahepatic tissues have been considered incapable of ketogenesis. Interestingly, recent studies have reported changes in gene and/or protein levels of Hmgcs2 in the hearts of mice and human patients (81, 87, 88). However, the role of Hmgcs2 in the normal heart or in the pathogenesis of cardiac disease is relatively unknown. In terms of ischemia-reperfusion injury, whether improvements in recovery are due to changes in ketone body oxidation or changes in oxidation of glucose or fatty acids is not clear and requires more extensive research.

Several studies from the same laboratory evaluated the effects of a low-carbohydrate diet (LCD) on tolerance to myocardial ischemia (105–108). The LCD diet consisted of 60% of the total calories from fat, 30% of total calories from protein, and 10% of the total calories from carbohydrates. Consumption of LCD was shown to induce a mild nutritional ketosis (108). Isolated hearts from lean and obese rats fed the diet for 2 weeks showed decreased recovery from low flow ischemia (105, 108). The 2-week LCD strategy also resulted in poor function, decreased survival, and increased arrhythmias in rats exposed to left anterior descending (LAD) ligation (107). One potential critique of these studies is the relatively short duration of the dietary intervention as the adaptation to KDs is purported to require 4–6 weeks (8). Since ketosis was relatively mild (~0.6 mM), perhaps, greater concentrations of serum ketone bodies would be required to cause positive adaptations. However, increasing the ketone body concentration to 1.2 mM did not improve the poor recovery in these animals (106, 108). Surprisingly, increasing ketone body concentrations in the perfusate did not appear to alter myocardial ketone body oxidation either in baseline or I-R conditions; however, there was a mild positive correlation between ketone body oxidation and recovery in all groups (106). Overall, these studies would suggest that a short-term (i.e., 2 weeks) diet of high fat and low carbohydrates would render the heart vulnerable to ischemic stress.

A recent study also evaluated the effectiveness of a KD on cardiac function following MI in mice (99). In this study, mice were fed a KD consisting of ~94% calories from fat and ~2% calories from carbohydrates for 4 weeks and then were subjected to LAD ligation. Four weeks following LAD ligation, infarct size was significantly greater and fractional shortening (FS) was significantly lower in mice fed the KD (99). MI hearts fed the KD also had lower protein content of glucose transporter 1 (Glut1) and hypoxia-inducible factor 1 alpha (HIF-*1*α) (99). The findings of this study suggest that elevations in serum ketone bodies have the potential to suppress glucose utilization in the ischemic heart, which may enhance myocardial injury.

In contrast to the above studies, fasting-induced ketosis (109) or long term LCKD (110) were associated with improved responses to ischemia in rats. Extreme fasting of 72 h increased β-OHB nearly 15-fold, which reduced infarct size and ventricular arrhythmias in Wistar rats subjected to occlusion of the LAD (109). Wistar rats fed a LCKD for 19-weeks demonstrated improved recovery following global ischemia compared to a control or high carbohydrate diet, which could be due to maintenance of mitochondrial number (110). Infusion of β-OHB to fed rats for 60 min prior to left coronary artery occlusion did not improve infarct size or functional recovery; however, β-OHB in rats fasted for 84 h led to reduced infarct size and higher functional recovery (111). Although the findings are promising, the severity of the fasting period or duration of the dietary intervention could be a bit impractical. Therefore, alternative methods of elevating ketone bodies may offer a more realistic treatment option.

Exogenous delivery of ketone bodies, especially β-OHB, may represent a suitable cardioprotective strategy for ischemia. Rat hearts reperfused with a glucose buffer containing a 5 mM ketone body concentration had improved LV contractility following 10 min of ischemia (27). Isolated mouse hearts provided with 3 mM β-OHB in the perfusate during reperfusion led to a significant improvement in functional recovery following 30 min of ischemia (112). These studies certainly highlight the potential of acute administration of β-OHB in supraphysiologic levels to improve recovery from ischemia. Mice implanted with an osmotic mini-pump with continuous delivery of β-OHB prior to reperfusion had reduced infarct size and preserved cardiac function (113). KE supplemented to rats, either immediately after or 2 weeks post, left coronary artery ligation resulted in an attenuation of pathological cardiac remodeling and improved ejection fraction compared to chow fed rats (114). Overall, the action of β-OHB or KE in the reperfused myocardium or ischemic myocardium may function via improved mitochondrial energetics (27, 114), reduced inflammation (112), and protection against oxidative stress (113). Certainly, additional investigations are required to further evaluate the therapeutic potential of ketone bodies from ischemic injury. In addition, studies examining long-term treatments would be more practical.

Recently, the use of sodium-glucose co-transporter 2 (SGLT2) inhibitors have been of increased interest in medicine and research due to decreased cardiovascular mortality and heart failure in diabetic patients (115). Although a variety of mechanisms have been proposed regarding the benefits of SGLT2 inhibitor on the heart (116), some studies suggest that SGLT2 inhibitors promote ketone body utilization (117, 118). In animal models, SGLT2 inhibitors are associated with beneficial outcomes in the non-diabetic ischemic heart (117–119). Administration of the SGLT2 inhibitor, dapagliflozin, prior to the onset of ischemia was associated with reduced infarct size and improved recovery in a rat model of *in-vivo* ischemia-reperfusion injury (119). Rats treated with empagliflozin for 10 weeks after MI induced by coronary artery ligation had improved cardiac function, an effect that was observed if treatment occurred 2 days prior to or 2 weeks after the surgical procedure (118). Of note, the positive functional outcomes with empagliflozin treatment were associated with elevated serum ketone bodies, upregulation of Bdh1 and Scot (Oxct1), and higher cardiac ATP content, suggesting that enhanced cardiac ketone body utilization was involved (118). In non-diabetic pigs, 2 months of empagliflozin treatment improved cardiac remodeling and LV function after proximal LAD occlusion, which was also associated with increased protein content of Bdh1 and Scot in the myocardium (117). Although these studies in animals are promising and appear to indicate that enhanced cardiac ketone body metabolism is mediating the response, additional work is clearly needed to evaluate the efficacy of SGLT2 inhibitors in ischemic heart disease.

## Conclusions And Perspectives

**Figure 2:**
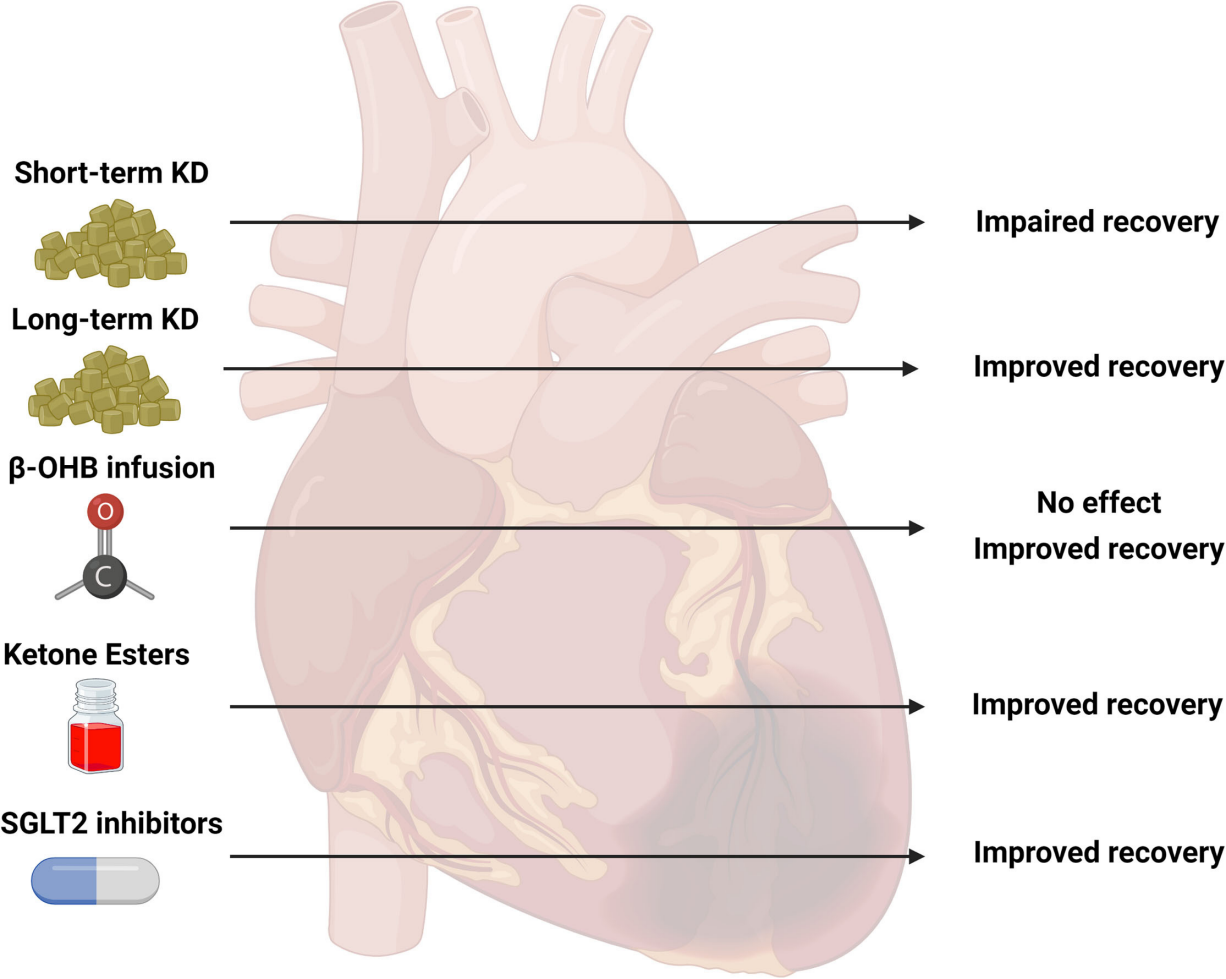
Summary of studies investigating ketone body metabolism in the ischemic heart. Various strategies to target ketone body metabolism in models of cardiac ischemia have been attempted including short-term ketogenic diets (KD, 2–4 weeks), long-term KD (19 weeks), infusion of beta-hydroxybutyrate (β-OHB), ketone ester (KE) supplementation, and sodium-glucose co-transporter 2 (SGLT2) inhibitors. The observed outcomes of these various interventions are presented as impaired, improved, or no effect. The current literature is limited on the effectiveness of these specific strategies: short-term KD (five studies), long-term KD (1 study), β-OHB infusion (four studies), KE supplementation (1 study), SGLT2 inhibitors (three studies). Created with BioRender.com.

Despite an increase in the number of studies focused on cardiac ketone body metabolism in pathological hypertrophy and heart failure, there seems to be a paucity of research elucidating the role of ketone body metabolism in the ischemic myocardium. Given the reported potential of ketone bodies to affect the use of fatty acids and glucose in the healthy myocardium, their delivery to the heart following bouts of ischemia could influence functional recovery. A summary of the findings from various strategies to enhance ketone body metabolism in models of ischemia are presented in Figure 2. Unfortunately, the current literature does not allow for definitive conclusions to be drawn, due to the relatively low number of studies in animal models. Studies investigating the effects of nutritional ketosis on recovery from ischemia appear to suggest that this strategy is detrimental. However, most of these studies are limited to short-term interventions. Physiological ketosis induced by fasting appears to be promising, but these animal studies seem impractical with fasting of 3 days or more. Delivery of exogenous ketone bodies (i.e., β-OHB) in *ex-vivo* perfused heart preparations have positive reports, but the concentrations used in these studies may be unrealistic compared to the *in-vivo* situation. Although the use of continuous β-OHB delivery via osmotic mini-pumps in mice demonstrates positive effects on functional recovery, the recent report that demonstrated an association between poor outcomes and high ketone body concentrations in humans following MI are equivocal. The use of SGLT2 inhibitors in non-diabetic hearts also appear to be promising. However, additional investigations to further evaluate all of these treatment strategies are warranted. For studies in animal models, careful attention should be paid to the concentrations used either in *in-vivo* or *ex-vivo* experimental conditions to ensure that the ranges of ketone bodies are within achievable concentrations consistent with nutritional or physiological ketosis. In human studies, a focus on the specific etiology of the disease (i.e., ischemic vs. non-ischemic) could help to delineate important contributions of ketone body metabolism to cardiac pathologies.

## Author Contributions

The author confirms being the sole contributor of this work and has approved it for publication.

## Funding

This work was supported by an AHA Institutional Research Enhancement Award from the American Heart Association (Grant Number: 20AIREA35080151).

## Conflict of Interest

The author declares that the research was conducted in the absence of any commercial or financial relationships that could be construed as a potential conflict of interest.

## Publisher's Note

All claims expressed in this article are solely those of the authors and do not necessarily represent those of their affiliated organizations, or those of the publisher, the editors and the reviewers. Any product that may be evaluated in this article, or claim that may be made by its manufacturer, is not guaranteed or endorsed by the publisher.
